# Major adverse cardiac events in patients with indeterminate high-sensitivity troponin testing and chest pain: a systematic review

**DOI:** 10.1007/s43678-026-01166-7

**Published:** 2026-05-14

**Authors:** Emma Helman, Emily Brossard, Jeremi Kolakowski, Abigail McIntosh, Chris Walsh, Shelley L. McLeod, Rohit Mohindra

**Affiliations:** 1 Faculty of Science, McGill University, Montreal, QC Canada; 2 Sinai Health, Toronto, ON Canada; 3 Schwartz/Reisman Emergency Medicine Institute, Toronto, ON Canada; 4 Division of Emergency Medicine, Department of Family and Community Medicine, Temerty Faculty of Medicine, University of Toronto, Toronto, ON Canada; 5 North York General Hospital, Toronto, ON Canada; 6 Division of Emergency Medicine, Department of Medicine, Temerty Faculty of Medicine, University of Toronto, Toronto, ON Canada; 7 Department of Public Health and Primary Care, University of Cambridge, Cambridge, England

**Keywords:** Cardiology, Chest pain, Troponin, cardiologie, douleur thoracique, troponine

## Abstract

**Objectives:**

Contemporary high-sensitivity cardiac troponin (hs-cTn) assays can detect very low levels of myocardial injury, leading many emergency department (ED) chest pain patients to exceed the 99th-percentile upper reference limit, despite low risk for myocardial infarction. This systematic review aimed to synthesize evidence quantifying the risk of MACE among ED patients with a single troponin concentration measurement above the 99th percentile reference limit but below diagnostic cutoffs for myocardial infarction.

**Methods:**

Electronic searches of MEDLINE, Cochrane Reviews, CINAHL and EMBASE (2002–2025) were conducted. Studies were included if outcomes for patients with indeterminate single hs-Tn measurements were reported or extractable. Patients with ECG findings suggestive of ST-segment elevation myocardial infarction (STEMI) or non-ST-segment elevation myocardial infarction (NSTEMI) were excluded. Two reviewers independently screened abstracts and extracted data. MACE was defined as acute myocardial infarction (MI), stroke, and cardiovascular mortality 30 days after index ED visit.

**Results:**

The search strategy yielded 709 potentially relevant citations. Eight studies were included: one randomized controlled trial, three prospective cohort, and four retrospective cohort studies. The incidence of MACE ranged from 0.3 to 14.8%. 46,066 of 129,060 eligible patients (40%) had a second test after an indeterminate result. Risk of bias was high for most observational studies, primarily due to information and incorporation bias, and randomized controlled trials had some concern for bias in the selection of the reported results, randomization process, and intended intervention.

**Conclusions:**

It remains unknown whether patients with an indeterminate hs-Tn result and no serial testing are at increased risk for MACE. More than half of such patients did not receive serial testing. The study demonstrates inconsistent approaches for managing indeterminate troponin results, underscoring the need for physicians to interpret indeterminate values cautiously and consider clinical assessment in low-risk chest pain patients.

**Supplementary Information:**

The online version contains supplementary material available at 10.1007/s43678-026-01166-7.

## Résumé

**Objectifs:**

Les tests contemporains de troponine cardiaque à haute sensibilité (hs-cTn) peuvent détecter des niveaux très faibles de lésion myocardique, ce qui conduit de nombreux patients souffrant de douleurs thoraciques aux urgences à dépasser la limite supérieure de référence du 99e percentile, malgré un faible risque d’infarctus du myocarde. Cette revue systématique visait à synthétiser les preuves quantifiant le risque de MACE chez les patients ED avec une seule mesure de concentration de troponine au-dessus de la limite de référence du 99e centile mais en dessous des seuils diagnostiques pour l’infarctus du myocarde.

**Méthodes:**

Des recherches électroniques dans MEDLINE, les revues Cochrane, CINAHL et EMBASE (2002-2025) ont été effectuées. Les études ont été incluses si les résultats pour les patients avec des mesures indéterminées de hs-Tn uniques étaient rapportés ou extractibles. Les patients dont l’ECG suggérait un infarctus du myocarde avec sus-décalage du segment ST (STEMI) ou sans sus-décalage du segment ST (NSTEMI) ont été exclus. Deux évaluateurs ont examiné indépendamment les résumés et extrait les données. MACE a été défini comme un infarctus du myocarde (MI) aigu, un accident vasculaire cérébral et une mortalité cardiovasculaire 30 jours après la visite index à l’urgence.

**Résultats:**

La stratégie de recherche a donné 709 citations potentiellement pertinentes. Huit études ont été incluses : un essai contrôlé randomisé, trois cohortes prospectives et quatre études de cohorte rétrospectives. L’incidence des MACE variait de 0,3 à 14,8 %. 46066 des 129060 patients éligibles (40 %) ont subi un deuxième test après un résultat indéterminé. Le risque de biais était élevé pour la plupart des études observationnelles, principalement en raison du biais lié à l’information et à l’incorporation, et les essais contrôlés randomisés avaient une certaine préoccupation quant au biais dans la sélection des résultats rapportés, du processus de randomisation et de l’intervention prévue.

**Conclusion:**

On ne sait toujours pas si les patients ayant un résultat indéterminé en hs-Tn et n’ayant subi aucun test en série présentent un risque accru de MACE. Plus de la moitié de ces patients n’ont pas reçu de tests en série. L’étude démontre des approches incohérentes pour gérer les résultats de troponine indéterminée, soulignant la nécessité pour les médecins d’interpréter prudemment les valeurs indéterminées et de considérer l’évaluation clinique chez les patients souffrant de douleurs thoraciques à faible risque.

## Clinician's capsule

**Table:** 

***What is known about the topic?***
Current guidelines recommend serial hs-Tn testing for ED patients with low-risk chest pain and a single indeterminate troponin test result.
***What did this study ask?***
What is the incidence of MACE in patients who have an indeterminate hs-Tn test result?
***What did this study find?***
MACE rate was highly variable, ranging from 0.3 - 14.8%, and only 40% of patients received an appropriate second test.
***Why does this study matter to clinicians?***
Standardized clinical procedures should be established regarding indeterminate troponin tests, focusing on evidence-based practices.

## Introduction

Despite being a leading cause of emergency department (ED) visits and hospitalization worldwide, most patients with chest pain are at low risk for acute coronary syndrome [1]. The introduction of high-sensitivity cardiac troponin (hs-cTn) has enabled more rapid and accurate evaluation of cardiac events in the emergency department, which may result in earlier discharge of low-risk patients [2]. Timely ED protocols reduce unnecessary hospital admissions and ED length of stay, prioritizing resources for life-threatening cases [3]. However, increased test sensitivity means more low-risk individuals exceed the 99th-percentile reference limit for myocardial injury. As a result, many clinical pathways now use assay-specific diagnostic cutoffs to rule in myocardial infarction, as recommended by the European Society of Cardiology Guidelines [4]. High-sensitivity troponin results are therefore interpreted across four functional ranges: undetectable, normal below the 99th percentile, indeterminate above the 99th percentile but below the rule-in threshold and elevated above the diagnostic threshold [3]. It is well understood that undetectable and normal values indicate low risk for MACE and elevated values suggest myocardial injury, while the indeterminate zone remains diagnostically ambiguous due to variability in assay-specific thresholds and follow-up protocols [5].

This systematic review aimed to synthesize evidence on the 30-day MACE risk among patients with an indeterminate hs-Tn concentration. Furthermore, we sought to quantify the number of patients who received an appropriate second test during further observation following an initial normal or indeterminate result. Lastly, we examined ED length of stay, 30-day hospital admission, and 30-day mortality for eligible patients with a second troponin after an initial indeterminate result. We hope to lay the groundwork for future investigation and decision-making regarding troponin protocols in the ED.

## Methods

### Data sources and search strategy

In consultation with the review authors, a research librarian (C.W.) with formal training in electronic literature searching conducted the literature searches in MEDLINE, Cochrane, EMBASE, and CINAHL from January 2002 to December 2025, as high-sensitivity troponin testing has only been in routine use for the last 20 years [6]. The reference lists of included articles were reviewed to identify additional relevant studies. A comprehensive search strategy (see Online Supplementary Material) included a combination of medical subject headings (MeSH) and free-text terms using various spelling and endings of key words. This report followed the Preferred Reporting Items for Systematic Reviews and Meta-Analysis (PRISMA) guidelines [7].

### Eligibility criteria and study selection

Studies were included if the study population comprised adult patients (≥ 18 years) presenting to an ED with a chief complaint of chest pain who underwent hs-cTn testing and had an indeterminate hs-cTn result. Studies conducted in chest pain units within EDs were also included. Our review included prospective and retrospective observational studies, case–control studies, and randomized controlled trials published in English. We excluded systematic reviews, case reports/series, expert opinions, letters to the editor, in-vitro studies, and studies assessing the cost-effectiveness of troponin testing or those evaluating conventional troponins. Studies in which serial troponin testing was mandated by a predefined diagnostic protocol for all participants were excluded, as they do not reflect decisions directed by clinicians for repeat testing following an initial indeterminate result. Patients who had ECG findings suggestive of STEMI/Acute Coronary Occlusion were excluded (see Online Supplementary Material).

Titles and abstracts were independently screened by two reviewers using a pre-established screening template. Full texts were then retrieved and assessed for inclusion. Any reviewer disagreement regarding inclusion was resolved by consensus or adjudicated by a third investigator if required.

### Outcome measures

A standardized data collection form was used to extract data pertaining to study design, setting, inclusion and exclusion criteria, and outcomes. The primary outcome of interest included MACE within 30 days of the index ED visit, including MACE on the index visit or during hospitalization. MACE was defined as acute myocardial infarction, stroke, or mortality. Secondary outcomes included the number of patients with an appropriate second troponin test, ED length of stay, 30-day hospital admission, and 30-day mortality. Despite overlapping troponin assays, the thresholds used to classify normal, indeterminate, and elevated results differed among included studies (see Online Supplementary Material). As a result, a generalized definition for an appropriate second test was applied as follows:

1. The first troponin result was detectable but not diagnostic for myocardial injury (i.e., normal or indeterminate).
2. The second troponin was done > 1 h after the first test.
3. The interpretation of the results is based on the rise or fall of troponin levels by > 20% of the initial measurement (i.e., delta troponin) as recommended by the Universal Definition of Myocardial Infarction [9].

Any inconsistencies within data extraction were discussed and resolved between reviewers. Authors were contacted for clarification of outcomes and methods if required.

### Risk of Bias assessment

Risk of bias for each randomized study was assessed using the Cochrane Collaboration’s Risk of Bias 2 tool [10]. For observational studies/non-randomized studies, this review utilized the Newcastle Ottawa Scale for Non-randomized Cohort studies [11]. Two authors conducted assessments, and discrepancies were resolved by discussion and consensus among authors.

### Data analysis

We were interested in examining patient outcomes across all studies. However, substantial heterogeneity in hs-cTn assay types and cutoffs across studies, as well as in outcome ascertainment (MACE event measured at index encounter versus after ED discharge), precluded any meaningful pooling of results. Thus, findings were synthesized narratively, and meta-analysis was not performed. Descriptive statistics were summarized using proportions, means, standard deviations, medians, and interquartile ranges, where appropriate.

## Results

**Fig. 1:**
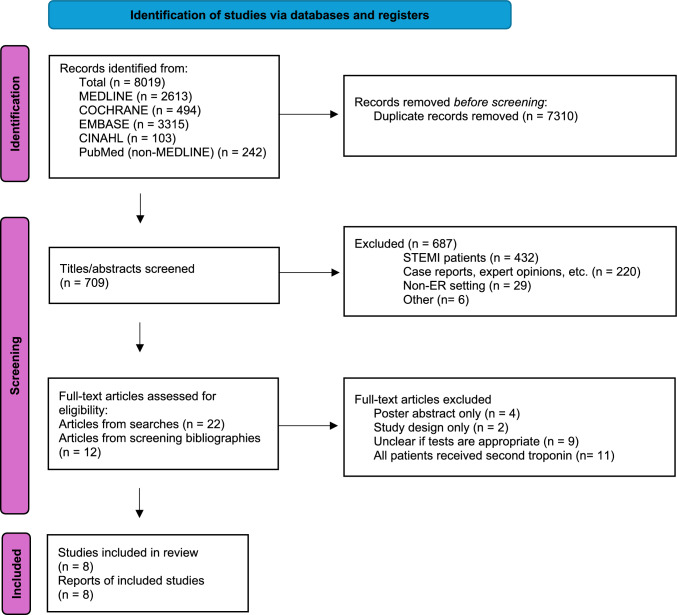
PRISMA flow diagram detailing article identification and screening process

The initial search strategy yielded 709 relevant citations after duplicates were removed (Fig. 1). After title and abstract screening, 34 articles underwent full-text review. Following full-text assessment, a total of eight studies were selected for inclusion in the systematic review (Table 1**)**. A total of 72,815 patients were included in the eight studies, with sample sizes ranging from 1,818 to 28,902 patients. There was one randomized controlled trial, three prospective cohort studies, and four retrospective cohort studies. Most studies were conducted in urban hospitals, and two studies were performed in chest pain units across Europe and Australia. Only three out of the eight studies described troponin cut-off values for serial troponin testing in their inclusion criteria. One study by Warren et al. (2024) was included for our secondary outcomes, despite not reporting our primary outcome of 30-day MACE risk [17]. In studies reporting multiple cohorts, we included only cohorts that represented patients with initial indeterminate results managed under clinical discretion. We excluded cohorts in which all patients underwent protocol-mandated repeat testing, as they do not reflect the clinical uncertainty raised in our research question. In one study, repeat testing was left to clinical decision, and some patients may not have received a second troponin.

**Table 1:** Summary of study characteristics of included articles

Author, year	Country	Study design	Setting	Sample size	Inclusion criteria	Exclusion criteria
Alazrag 2024 [12]	Australia	Cohort Retrospective	Urban	2,738	Patients with suspected acute coronary syndrome, hs-TnT measurements (most > 1, hs-TnT levels > 15 ng/L but did not meet MI criteria based on 4th UDMI)	Patients classified as MI ‘ruled-in’ or MI ‘ruled-out’, first sample hs-TnT had haemolysis, only 1 hs-TnT, incomplete data for patient outcomes
Chew 2019 [13]	Australia	Randomized controlled trial	Urban	3,378	Age ≥ 18 yrs, presenting to ED with suspected acute coronary syndrome or chest pain and baseline ECG not definitive for coronary ischemia	Suspected non-cardiac cause, transfer from another hospital, suspected acute coronary syndrome within 30 days of last presentation, permanent dialysis required, unable to complete clinical history questionnaire
Cohen 2023 [14]	Israel	Cohort, Prospective	Urban	11,477	Suspected NSTE-acute coronary syndrome, single hs-cTnT < 5 ng/L or initial value ≥ 5 ng/L but < 14 ng/L and increments of 3 ng/L at 1 h, 4 ng/L at 2 h for subsequent tests	STEMI, referred to ED for further evaluation, respiratory complaints at presentation, suspected silent ischaemia, type II MI, initial hs-cTnT > 5 ng/L but < 14 ng/L for single troponin
Keller 2011 [5]	Germany	Cohort, Prospective	CPU	1,818	Age 18–85, with acute angina pectoris/equivalent symptoms	Major surgery or trauma within 4 weeks, pregnancy, intravenous drug abuse, anemia (hemoglobin < 10 g/dL)
Pareek 2023 [15]	Denmark	Cohort, Retrospective	Urban	28,902	Patients discharged with MI, unstable angina, suspected MI, or chest pain, hs-cTnT testing > 1 h and < 7 h apart during hospitalization	Patients with only one sample or 2 samples drawn outside of 1–7 h, patients missing hemoglobin/creatinine measurement
Pareek 2024 [16]	Denmark	Cohort, Retrospective	Urban	10,377	Patients hospitalized for suspected acute coronary syndrome with 2 hs-TnI measurements separated by 1–7 h, during a first-time hospitalization for MI, unstable angina, observation for suspected MI, or chest pain	Missing hemoglobin or creatine measurements, incomplete patient data for troponin-I measurements or not drawn within desired timeframe
Twerenbold 2018 [8]	Switzerland, Italy, Spain, Poland, Czech Republic, Germany	Cohort, Prospective	Urban, Academic	4,368	Adult patients presenting to ED with symptoms suggestive of MI, including acute chest discomfort, angina pectoris	Patients presenting with STEMI, missing or invalid troponin measurement
Warren 2024 [17]	United States	Cohort, Retrospective	Urban	9,757	Patients with at least 1 cTn test result presenting to the ED	Patients who left after assessment, missing a disposition order to either admit or discharge, had an ED LOS > 72 h, (thought to represent inaccuracies in the dataset), or did not have cTn test resulted

Where: *hs-Tn* high-sensitivity troponin, *NSTE* non-ST-elevation, *STEMI* ST-segment elevation myocardial infarction, *UDMI* Universal Definition of Myocardial Infarction

Risk of bias was high for most observational studies, primarily due to low or absent comparability of the cohort based on the design or analysis (Table 2**)**. The randomized controlled trial has some concern for bias in three out of five domains, including the randomization process, intended interventions, and selection of reported results (Table 3).

**Table 2:** Risk of bias assessment for cohort studies using the Newcastle–Ottawa Scale (NOS). Risk of bias is assessed across three broad categories: the selection of study groups, the comparability of the groups, and the ascertainment of the outcome of interest. Each study can be rewarded a maximum of nine stars total, with nine indicating a higher quality study

Study label	Study type	Selection (Total Stars) *maximum 4 stars*	Comparability (Total Stars) *maximum 2 stars*	Outcome (Total Stars) *maximum 3 stars*	Total stars *maximum 9 stars*
Twerenbold 2018	Cohort, Prospective	4	0	3	7
Alazrag 2024	Cohort, Retrospective	4	1	3	8
Cohen 2023	Cohort, Prospective	3	0	3	6
Keller 2011	Cohort, Prospective	4	0	3	7
Pareek 2023	Cohort, Retrospective	4	1	3	8
Pareek 2024	Cohort, Retrospective	4	1	3	8
Warren 2024	Cohort, Retrospective	4	1	3	8

**Table 3:** Risk of bias assessment for randomized controlled trial study assessed using the RoB-2 risk of bias tool [10]

**Study label**	**Outcome**	**D1**	**D2**	**D3**	**D4**	**D5**	**Overall**
Chew 2019	MACE	Some concern	Low risk	Low risk	Low risk	High risk	High risk

Risk of bias is assessed where: *D1* randomization process, *D2* deviations from intended interventions, *D3* missing outcome data, *D4* measurement of outcome, *D5* selection of the reported result

Across all studies, 1,626 of the 31,321 patients (5.2%) with secondary troponin testing after an indeterminate or negative test were diagnosed with MACE within 30 days (Table 4). Most studies did not distinguish between MACE on the index ED encounter and MACE after ED discharge. Notably, the study by Chew (2019) did not include MACE at index and only documented MI after participants were discharged [13]. In contrast, Cohen (2023) recorded MI events on ED index visits [14]. The incidence of MACE within studies ranged from 0.3–14.8%, with a mean of 4.9% (SD = 0.06). Three studies lacked sufficient data to calculate MACE within 30 days, even after contacting the study authors, yet addressed secondary outcomes [5, 15, 17] (Table 4). Our secondary outcome was the rate of appropriate serial testing in patients with indeterminate hs-cTn. Of the 129,060 eligible patients, 46,066 (40%) had appropriate second troponin testing (SD = 23%), as defined previously.

**Table 4:** Summary of primary and secondary study outcomes of interest

**Author, Year**	**30-day acute coronary event in patients with myocardial injury**	**Event on index encounter or after ED discharge**	**Number of patients with appropriate second troponin**	**ED length** **of stay**	**Hospital admission** **within 30 days**	**30-day mortality**
Alazrag, 2024	17 (3.1%)	Not distinguished	NA	8.2 h	65 (11.9%)	12 (2%)
Chew, 2019	18 (5.8%)	After discharge	308 (18.7%)	5.6 h	15 (0.9%)	6 (0.04%)
Cohen, 2023	6 (0.3%)	Index encounter	3,918 (17.2%)	5.0 h	3,775 (32.9%)	2 (0.05%)
Keller, 2011	NA	NA	1,260 (69.3%)	-	-	20 (1.1%)
Pareek, 2023	NA	NA	29,363 (55.9%)	-	-	2,169 (7.5%)
Pareek, 2024	1,538 (14.8%)	Not distinguished	5,756 (14.9%)	-	-	1,420 (6.9%)
Twerenbold, 2018	11 (0.5%)	Not distinguished	1107 (25.3%)	-	-	2 (0.05%)
Warren, 2024	NA	NA	2999 (65.3%)	2.0 h	-	-

*NA* results were not available from the primary manuscript, supplementary material, or after contacting the authors. Event on index encounter indicates if the study distinguished between MACE occurring on index ED visit or after the ED discharge

The average ED length of stay was 5.2 h (SD = 2.54), with a range of 2.0–8.2 h in four included studies. Across three studies, 3,855 patients of 13,666 (28%, SD ± 16%) eligible patients were admitted to the hospital within 30 days of index ED visit. A total of 3,631 of 60,726 eligible patients (6%, ± 3%) across seven studies died within 30 days of index ED visit.

## Discussion

### Interpretation of findings

Among the eight studies included in the primary outcome, the proportion of patients with MACE at 30 days ranged from 0.3% to 14.8%, a range that is too variable to estimate a distinct risk. This variation is likely driven by differences in population characteristics and patient heterogeneity. Previous literature suggests a MACE rate of 0.5% in low-risk patients (i.e., with troponin below the limit of detection), yet a 15% MACE rate for patients with a single indeterminate troponin result [18, 19]. Given the significant heterogeneity in MACE rates among included studies, it is difficult to draw exact conclusions on MACE risk; however, given the high potential risk of adverse cardiac events, indeterminate patients should be treated with increased clinical vigilance.

Despite overlap in hs-cTn assays used, the included studies used varying measurements to differentiate normal, indeterminate, and elevated troponin results, reflecting ambiguity in ED protocols (see Online Supplementary Material). Additionally, the risk of bias was generally high among observational studies, and the randomized controlled trial raised similar concerns for bias, further limiting the strength of these findings. Notably, 60% of patients with an indeterminate result did not undergo appropriate serial hs-cTn testing, emphasizing the need for clarified ED protocols.

### Comparison to previous studies

Currently, there are very few systematic reviews investigating the risk of MACE for patients following an initial indeterminate hs-cTn result. Despite limited comparative data, one large meta-analysis by Chapman et al. (2017) revealed that patients with very low hs-cTn concentrations at presentation (< 5 ng/L) have an approximate risk of 0.5% for MACE at 30 days and low 1-year mortality [20]. In contrast, our findings suggest the true risk of MACE remains uncertain for patients with indeterminate troponin results. Joyce et al*.* (2023) emphasized the effectiveness of single hs-cTn testing for patients with troponin levels well below the 99th percentile, but note that across 30 cohort studies, 50% of missed cases occurred when a single test was conducted too early after symptom onset, leading to inappropriate discharge [21]. A similar review by Potomac and Diercks (2019) further underscored the difficulty of accurately assessing MACE risk without integrating medical history and physical examination findings [22].

Among the included studies in this review, the study by Alazrag et al*.* (2024) was particularly relevant as it investigated the management and outcomes of patients presenting to the ED with symptoms suggestive of myocardial injury [12]. The study found that 20–30% of patients were neither ruled-in nor ruled-out for MI, thus were deemed to require further observation, corresponding to our definition of indeterminate troponin values. These patients had a MACE rate of 3.1%. The observation group was defined as patients whose baseline hs-TnT was 15-49 ng/L, with a change less than 3 ng/L/h or a relative change between 10%-50% [12]. The 1- and 5-year mortality rates of patients in the observation group were higher than those with confirmed type I MI [12], emphasizing the need for more evidence-based protocols for patients with indeterminate results.

### Strengths and limitations

This systematic review follows standard PRISMA guidelines, and the search strategy was designed by an experienced clinical information specialist, ensuring no relevant studies were missed. We performed a comprehensive risk of bias assessment for included studies, and our review asked a clinically important question in emergency medicine that has been historically underexplored.

This systematic review has several important limitations. Only English-language articles were included in our reviews. In some studies, it was unclear whether troponin testing took place in the ED or in inpatient settings. Authors were contacted in this case, but they were unable to provide further clarification. Variation in standard procedures across countries may also lead to discrepancies when comparing these studies. For example, in Denmark, chest pain patients are usually always admitted. In contrast, in Germany, the recent development of certified Chest Pain Units complicates admission protocols for patients with low-risk chest pain. It is important to note the distinction between MACE diagnosed during the index ED visit and events occurring after discharge, as failure to distinguish between these pathways risks introducing misclassification and limits the comparability of the event rates across studies. Most studies were unable to distinguish between these rates, or only reported events at one of these timepoints. This may have contributed, in part, to the large variation across MACE rates observed. Coronary revascularization is commonly included as a component of MACE in chest pain literature; however, it was not used as an outcome in this review because only two included studies reported revascularization rates, precluding meaningful comparison across studies [13, 14]. Furthermore, there was no standardized method for physicians to order serial troponins following intermediate hs-cTn results. The ESC recommends serial measurements of hs-cTn levels over 1 to 3 h after presentation and using peak hs-cTn level and changes in hs-cTn over time (delta hs-cTn) to guide the decision to discharge a patient [4]. Still, there is no consensus on the specific algorithm that should be employed by emergency physicians, which ultimately leads us to our primary question, and creates inherent limitations during data abstraction. Most included studies did not provide any rationale for serial versus single troponin testing. Risk scores may influence this decision-making; however, use of risk scores was generally underreported. Chew et al. (2019) noted that clinical information required for the calculation of commonly used risk scores was collected and available to clinicians [13]. It is unclear whether risk scores influenced approaches to troponin testing, but they should not substantially affect decisions about serial testing.

### Clinical implications

The lack of a standardized approach for managing indeterminate troponin results in clinical practice poses a significant risk for inconsistent care and potential patient harm. The study demonstrates a risk for MACE after an indeterminate troponin test, yet limited clarity on how practicing physicians should interpret and act. It is therefore imperative that clinicians remain aware of the potential risk for patients presenting to the ED with chest pain and remain vigilant.

### Research implications

Future studies should focus on establishing optimal, evidence-based testing pathways for patients with indeterminate hs-cTn measurement. Examining outcomes in this patient population would help to refine our understanding of MACE risk and inform ED management strategies. Additionally, incorporating patient perspectives into decision-making processes will be essential for developing clinically effective, patient-centered guidelines for troponin interpretation.

## Conclusion

This systematic review highlights a critical gap in understanding clinical outcomes for ED patients with low-risk chest pain and indeterminate hs-Tn results. Observed 30-day MACE rates were highly variable, ranging from 0.3–14.8%. This suggests an area of high potential risk, yet current evidence does not permit reliable risk estimation for this patient population. Furthermore, only 40% of eligible patients received an appropriate second test, indicating substantial under-utilization of serial testing. Given the nature of MACE risk, clinicians should maintain increased vigilance and avoid relying on single indeterminate troponin results. When serial testing is available, patients should undergo repeat hs-cTn testing and reassessment before discharge. In settings where this is not feasible, clinicians should interpret indeterminate results with caution, and use structured risk scores and close follow-up to minimize the likelihood of adverse outcomes. Our findings highlight the need for more rigorous evidence to guide clinical practice and inform guidelines on how to manage indeterminate hs-cTn results.

## Supplementary Information

Below is the link to the electronic supplementary material.

Supplementary Material File 1 (DOCX 144 KB)

## Data Availability

No new data was generated or analyzed in this review. All data supporting findings are available within the article and supplementary materials. Additional details are available from authors upon request.
