# Supplementary material for: Major adverse cardiac events in patients with indeterminate high-sensitivity troponin testing and chest pain: a systematic review
Source: CJEM. 2026 May 14;28(7):642–50. doi: 10.1007/s43678-026-01166-7 (PMC13337960; doi:10.1007/s43678-026-01166-7)
Supplement: Supplementary file 1 — Supplementary Material File 1 (DOCX 144 KB) [file 43678_2026_1166_MOESM1_ESM.docx]

**Appendix A. Hs-Tn immunoassay details and study-specific criteria defining indeterminate results.** This table summarizes the assay type, reported limit of detection (LOD), reported upper reference limit (URL), and the measurement criteria used across included studies to identify patients aligning with our definition of indeterminate results (i.e., above the 99th percentile upper reference limit but below assay-specific diagnostic thresholds).

| **Author, Year** | **Assay Type, Reported LOD, Reported 99th percentile URL** | **Study-Specific Criteria for Indeterminate hs-Tn Result** |
| --- | --- | --- |
| Alazrag, 2024 | Roche Diagnostics, unspecified generation, URL: 14 ng/L | Initial troponin between 15–49 ng/L with a change <3 ng/L/h or an absolute change between 5–10 ng/L or a relative change of 10–-50%. |
| Chew, 2019 | Roche Diagnostics, transition between Elecsys 4th- and 5th-generation hs-cTnT assay, LOD: 5 ng/L, URL: 14 ng/L | Initial troponin between 13–51 ng/L with a 1–hour change <5 ng/L or initial troponin ≤12 ng/L with a 1–hour change of 3–4 ng/L. |
| Cohen, 2023 | Roche Diagnostics, Elecsys 4th-generation hs-cTnT assay, LOD: 5 ng/L, URL: 14 ng/L | A single initial value <5 ng/L or an initial value between 5–14 ng/L with an increment of <3 ng/L in a subsequent test. |
| Keller, 2011 | Abbott Diagnostics, ARCHITECT STAT High Sensitive Troponin-I, LOD: 3.4 ng/L, URL: 29.9 ng/L | Not specified |
| Pareek, 2023 | Roche Diagnostics, Elecsys 5th-generation hs-cTnT assay, LOD: 5ng/L, URL: 13.5 ng/L | Initial troponin <14 ng/L and a subsequent test 1–7 hours later |
| Pareek, 2024 | Siemens Healthcare Diagnostics, TnI Flex® reagent assay URL: 45 ng/L | Initial troponin <45 ng/L and a subsequent test 1–7 hours later |
| Twerenbold, 2018 | Roche Diagnostics, Elecsys 5th-generation hs-cTnT assay, LOD: 5ng/L, URL: 13.5 ng/L and Abbott Diagnostics, ARCHITECT STAT High Sensitive Troponin-I, LOD: 3.4 ng/L, URL: 29.9 ng/L | Initial troponin <5 ng/L with a 1–hour change >2ng/L or an initial troponin between 5–52 ng/L with a 1–hour change <6 ng/L |
| Warren, 2024 | SiemensHealthcare Diagnostics, Centaur hs-cTnI assay, LOD: 2.5 ng/L, 99th percentile URL: 58 ng/L for males and 39 ng/L for females | Not specified |

**Appendix B. Database search strategies for all databases**. The following outlines the detailed search strategies used in MEDLINE, CINAHL, Embase, and the Cochrane Library as part of the literature review. Searches were conducted on April 25, 2025.

**Ovid MEDLINE (R) / Cochrane**

ALL <2002 to April 25, 2025>

1 Troponin I/

2 troponin I.ti,ab.

3 Troponin/

4 "troponin".ti,ab.

5 (troponin adj3 (test* or concentration* or draw* or report* or measur* or value* or threshold* or level* or

elevat*)).ti,ab.

6 (troponin adj3 repeat*).ti,ab.

7 (troponin adj3 sensitiv*).ti,ab.

8 Chest Pain/

9 Myocardial Infarction/

10 Coronary Artery Disease/ or Acute Coronary Syndrome/ or Coronary Disease/

11 Heart Diseases/

12 Myocytes, Cardiac/

13 Angina, Unstable/ or Angina Pectoris/

14 (chest adj2 pain*).ti,ab.

15 (myocard* adj2 (stroke* or infarction or damag* or disease*)).ti,ab.

16 (heart adj1 (disease* or attack* or failure*)).ti,ab.

17 (cardiac adj2 myocyte*).ti,ab.

18 (coronary adj2 (disease* or syndrome*)).ti,ab.

19 angina.ti,ab.

20 (interm* or medium* or elevat* or serial or non-diagnostic or "non diagnostic" or modest).ti.

21 troponin c/ or troponin t/

22 repeat*.ti.

23 1 or 2 or 3 or 4 or 5 or 6 or 7 or 21

24 8 or 9 or 10 or 11 or 12 or 13 or 14 or 15 or 16 or 17 or 18 or 19

25 23 and 24

26 20 or 22

27 25 and 26

28 exp animals/ not humans.sh.

29 27 not 28

**CINAHL**

S1 (MH "Troponin")

S2 TI troponin OR AB troponin

S3 TI ( (troponin N3 (test* or concentration* or draw* or report* or measur* or value* or threshold* or level* or elevat*)) ) OR AB ( (troponin N3 (test* or concentration* or draw* or report* or measur* or value* or threshold* or level* or elevat*)) )

S4 TI (troponin N3 repeat*) OR AB (troponin N3 repeat*)

S5 TI (troponin N3 sensitiv*) OR AB (troponin N3 sensitiv*)

S6 (MH "Chest Pain+")

S7 (MH "Myocardial Infarction+")

S8 (MH "Coronary Arteriosclerosis")

S9 (MH "Acute Coronary Syndrome")

S10 (MH "Heart Diseases+")

S11 (MH "Myocytes, Cardiac")

S12 (MH "Angina, Unstable")

S13 (MH "Angina Pectoris+")

S14 TI (chest N2 pain*) OR AB (chest N2 pain*)

S15 TI ( (myocard* N2 (stroke* or infarction or damag* or disease*)) ) OR AB ( (myocard* N2 (stroke* or infarction or damag* or disease*)) )

S16 TI ( (heart N1 (disease* or attack* or failure*)) ) OR AB ( (heart N1 (disease* or attack* or failure*)) )

S17 TI (cardiac N2 myocyte*) OR AB (cardiac N2 myocyte*)

S18 TI ( (coronary N2 (disease* or syndrome*)) ) OR AB ( (coronary N2 (disease* or syndrome*)) )

S19 TI angina OR AB angina

S20 TI (interm* or medium* or elevat* or serial or non-diagnostic or "non diagnostic" or modest)

S21 TI repeat*

S22 S1 OR S2 OR S3 OR S4 OR S5

S23 S6 OR S7 OR S8 OR S9 OR S10 OR S11 OR S12 OR S13 OR S14 OR S15 OR S16 OR S17 OR S18 OR S19

S24 S22 AND S23

S25 S20 OR S21

S26 S24 AND S25

S27 S24 AND S25

| **Embase Classic+Embase** | |
| --- | --- |
| **#** | **Search Statement** |
| 1 | exp troponin I/ |
| 2 | troponin I.ti,ab. |
| 3 | exp troponin/ |
| 4 | troponin.ti,ab. |
| 5 | (troponin adj3 (test* or concentration* or draw* or report* or measur* or value* or threshold* or level* or elevat*)).ti,ab. |
| 6 | (troponin adj3 repeat*).ti,ab. |
| 7 | (troponin adj3 sensitiv*).ti,ab. |
| 8 | exp thorax pain/ |
| 9 | exp heart infarction/ |
| 10 | exp coronary artery disease/ |
| 11 | exp acute coronary syndrome/ |
| 12 | exp heart disease/ |
| 13 | exp cardiac muscle cell/ |
| 14 | exp unstable angina pectoris/ |
| 15 | exp angina pectoris/ |
| 16 | (chest adj2 pain*).ti,ab. |
| 17 | (myocard* adj2 (stroke* or infarction or damag* or disease*)).ti,ab. |
| 18 | (heart adj1 (disease* or attack* or failure*)).ti,ab. |
| 19 | (cardiac adj2 myocyte*).ti,ab. |
| 20 | (coronary adj2 (disease* or syndrome*)).ti,ab. |
| 21 | angina.ti,ab. |
| 22 | (interm* or medium* or elevat* or serial or non-diagnostic or "non diagnostic" or modest).ti. |
| 23 | exp troponin T/ |
| 24 | repeat*.ti. |
| 25 | 1 or 2 or 3 or 4 or 5 or 6 or 7 or 23 |
| 26 | 8 or 9 or 10 or 11 or 12 or 13 or 14 or 15 or 16 or 17 or 18 or 19 or 20 or 21 |
| 27 | 25 and 26 |
| 28 | 22 or 24 |
| 29 | 27 and 28 |
| 30 | limit 29 to human |

**Appendix C. Study information on ECG features suggestive of NSTEMI.** Clarification of methods for each study included related to ECG features and whether patients with NSTEMI were included or excluded.

| **Author, Year** | **ECG Features/Criteria** |
| --- | --- |
| Alazrag, 2024 | Did not explicitly mention ECG criteria in exclusion or inclusion criteria |
| Chew, 2019 | Included patients whose initial ECG assessment did not provide a high diagnostic likelihood for MI; i.e. baseline ECG interpreted as not definitive for coronary ischemia. |
| Cohen, 2023 | Did not explicitly mention ECG criteria in their exclusion or inclusion criteria. |
| Keller, 2011 | Did not report ECG features or mention it in exclusion or inclusion criteria. |
| Pareek, 2023 | No mention of use of ECG assessment in the low-risk population. |
| Pareek, 2024 | No mention of ECG assessment in the low-risk population. |
| Twerenbold, 2018 | Patients presenting with ST-segment elevation myocardial infarction were excluded, but patients with symptoms suggestive of NSTEMI were included in this study. It should be noted that these patients were recruited for the study based on suggestive symptoms such as acute chest discomfort, and/or angina pectoris rather than ECG characteristics. |
| Warren, 2024 | No information regarding ECG characteristics or exclusion of patients with NSTEMI/STEMI was reported. |
